# Neural Dynamics of Learning Sound—Action Associations

**DOI:** 10.1371/journal.pone.0003845

**Published:** 2008-12-03

**Authors:** Adam McNamara, Giovanni Buccino, Mareike M. Menz, Jan Gläscher, Thomas Wolbers, Annette Baumgärtner, Ferdinand Binkofski

**Affiliations:** 1 NeuroImage Nord, Department of Neurology, UKSH, Luebeck, Germany; 2 Department of Psychology, University of Surrey, Guildford, United Kingdom; 3 Department of Neurosciences, Universita Degli Studi di Parma, Parma, Italy; 4 NeuroImage Nord, Department of Systems Neuroscience, University Medical Centre Hamburg Eppendorf, Hamburg, Germany; University of St. Andrews, United Kingdom

## Abstract

A motor component is pre-requisite to any communicative act as one must inherently move to communicate. To learn to make a communicative act, the brain must be able to dynamically associate arbitrary percepts to the neural substrate underlying the pre-requisite motor activity. We aimed to investigate whether brain regions involved in complex gestures (ventral pre-motor cortex, Brodmann Area 44) were involved in mediating association between novel abstract auditory stimuli and novel gestural movements. In a functional resonance imaging (fMRI) study we asked participants to learn associations between previously unrelated novel sounds and meaningless gestures inside the scanner. We use functional connectivity analysis to eliminate the often present confound of ‘strategic covert naming’ when dealing with BA44 and to rule out effects of non-specific reductions in signal. Brodmann Area 44, a region incorporating Broca's region showed strong, bilateral, negative correlation of BOLD (blood oxygen level dependent) response with learning of sound-action associations during data acquisition. Left-inferior-parietal-lobule (l-IPL) and bilateral loci in and around visual area V5, right-orbital-frontal-gyrus, right-hippocampus, left-para-hippocampus, right-head-of-caudate, right-insula and left-lingual-gyrus also showed decreases in BOLD response with learning. Concurrent with these decreases in BOLD response, an increasing connectivity between areas of the imaged network as well as the right-middle-frontal-gyrus with rising learning performance was revealed by a psychophysiological interaction (PPI) analysis. The increasing connectivity therefore occurs within an increasingly energy efficient network as learning proceeds. Strongest learning related connectivity between regions was found when analysing BA44 and l-IPL seeds. The results clearly show that BA44 and l-IPL is dynamically involved in linking gesture and sound and therefore provides evidence that one of the mechanisms required for the evolution of human communication is found within these motor regions.

## Introduction

Approaching communication from a strictly neuro-biological perspective, speech and gestures may be conceptually regarded as biologically pure signs, distinct in that they require no tool for production. Two things are common and inherent to all such signs. Firstly, to be communicated they must be replicable i.e., imitable. This means that irrespective of whatever other regions of the brain contain representation relevant to each sign, a motor representation is inherently included. Inherent because one must use muscles to speak a word, or make a gesture. Movement is pre-requisite to communication. Secondly, the meaning of these signs has to be learnt. *Initial* learning of the signs must entail linkage of sensory, proprio-sensory and internal state representations to the communicative motor representation. Economy and efficiency is a fundamental principle of biological systems [1], reduction in redundancy of processing would be attained by having the locus for initial binding of multimodal representations within neural regions pre-requisite to all, i.e., within the motor system. Specifically, those regions within the motor system that are known to be involved in performing complex gestures of the primary affectors, the hand and orofacial muscles such as the ventral pre-motor cortex [2]–[6].

Implicit to all theories of language evolution is that the neural substrate of a motoric action involved in communicating a concept must somehow be linked to the neural substrates encoding that concept. i.e., that the signifier is linked to the signified [7]. To achieve this in speech, the brain must be able to dynamically associate arbitrary sounds to motor activity involved in gesture. Given the heavy weight of auditory and visual stimulus in human communication, one may anticipate that linking arbitrary sounds to the motor sequences for conducting gesture is an important mechanism required for the evolution of human communication. We test the motor system, specifically vPMC, Brodmann Area 44 (BA44) to identify if it is able to carry out initial associative learning of multimodal stimuli as required for learning communicative acts.

Our deductive reasoning converges with the data from biological experiments describing BA44 as part of the human homologue of monkey area F5 [8], [9], a main component of a system coined as the mirror neuron system (MNS) [9], [10]. The MNS has been proposed as playing a key role in the evolution of language [11]–[15] and subsequently to social cognition at an even more general level [16].

The motor system's role in effecting understanding between conspecifics has been identified in other ways. Several recent studies demonstrated that seeing and hearing words recruits parts of the motor system actually involved in the production of these same words [17]–[19]. Additionally, words such as ‘kick’ or ‘punch’ activate appropriate limb muscles [20]. These results and imaging data [19], support the notion of a “phonological resonance” allowing for an automatic recruitment of motor structures active during speech production and also during speech processing. Not only words, but also meaningful sounds can activate the motor system [21]–[25]. This motor resonance independent of semantics dovetails with Liberman's, ‘Motor Theory of Speech Perception’ [26]–a theory of speech which posits phonemes as the interface between the perception and the production of language.

BA44 is also active during the observation of mouth and hand actions, either when they are object directed or they have a communicative character [27]–[29]. A pre-requisite to posit the role of the motor system, and specifically of vPMC, in the development of language is that within this region, unrelated sounds and the objects or the actions they refer to may be combined. Evidence that the formation of sound-action pairs is mediated by vPMC is still lacking.

In the current study we specifically addressed the issue of whether BA44, or a part of it, is involved in the formation of links between sounds and actions. To avoid contamination by previous learning related to objects or actions already represented in the brain, we choose to combine novel sounds with non-object directed meaningless gestures. If BA44 is mediating the association of specific representations to sounds, then we hypothesized that changes in BOLD signal would occur within this region over learning. Additionally, that this effect would enable us to identify other structures with which BA44 communicates. The former was evaluated by correlation of the learning of sound-gesture associations with the BOLD signal, the latter question was addressed by application of functional connectivity analysis in order to identify brain areas working together in the dynamic process of learning sound-gesture associations.

## Materials and Methods

12 healthy right-handed volunteers (6 females, mean age 27.25 years) entered the study. Each of the subjects gave written informed consent and the study was approved by the local (Hamburg Board of Physicians) ethics committee acting in accord with the declaration of Helsinki.

**Figure 1:**
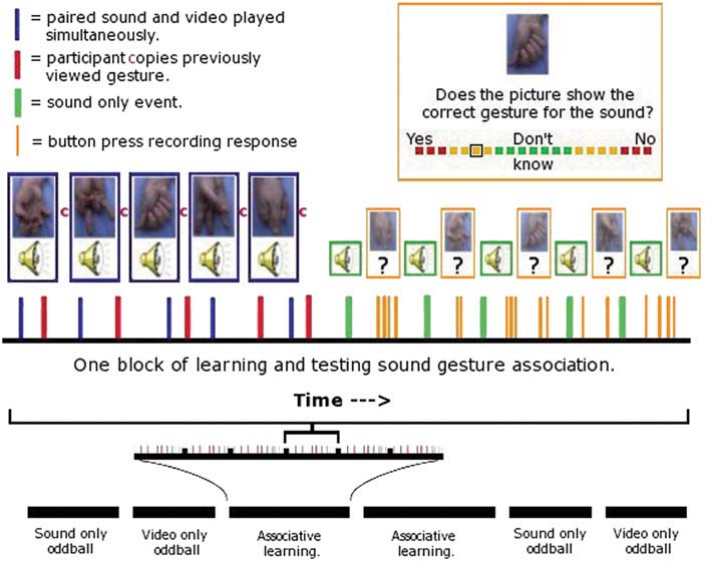
Presentation of the experimental design. Prior to, and post associative learning, gesture and sound stimuli are presented unpaired to the subjects to familiarize them with the stimuli and to reduce habituation effects, (see Methods). During ‘associative learning’ data acquisitions, participants observe hand actions whilst hearing the paired sound stimulus (blue). After an interval the word ‘copy’ is presented (red) and the participant imitates the action just seen. Next, five test trials occur. For each test trial, first the sound only is presented (green 1.5 sec). After this, a still image of one of the actions is presented. Participants rate whether the picture matches the sound or not on a colour coded 40 point visual analogue scale. No feedback is given.

While lying in the MRI scanner, subjects were asked to learn associations between meaningless hand gestures and synthetic sounds. For this purpose we developed five meaningless hand gestures, presented as 1.5 s videos, and 5 synthetic meaningless sounds. One specific gesture had to be associated with one specific sound. Prior to the association learning task, familiarisation of stimuli was conducted using an oddball detection task for gestures as well as sounds. Subjects were shown the oddball target, (another hand gesture and sound) prior to scanning and told to press the button when they saw/heard the target. The oddball targets (n = 3) were then randomly placed in a stream of events consisting of the test stimuli (n = 30; 6 repeats of each of the 5 stimuli). Both familiarisation periods were then carried out whilst scanning, (Figure 1). In order to inscribe the observed gestures into the participant's motor repertoire, volunteers were required to imitate them after viewing each stimulus. The experimental paradigm is presented in Figure 1 and fully described in the legend. The key event in the paradigm, is the ‘sound only’ event during the test blocks of the learning sessions. These events occurred regularly throughout the learning process, allowing us to identify parametric modulation of response to the sound by learning [30].

Video clips (see Supplementary Materials ‘Video S1’) were all recorded under identical lighting conditions against a blue background cloth. Each of the video clips showed the performance of a meaningless hand gesture. Each gesture was begun from the same relaxed, right hand position and was completed within 1 second of the video onset. The final hand position of the gesture was then held for the remainder of the 1.5 s duration of the video. Sounds (see Supplementary Materials ‘Video S1’) were two fixed sine waves of 450 Hz and 850 Hz and three frequency modulated 500 Hz sine waves creating sounds with constant undulations or gradually increasing or decreasing undulations in frequency, (“Goldwave”, www.goldwave.com). Presentation of the videos was achieved by presenting a succession of 45 centrally located still images for 33 ms (no gap), which provided smooth video like movement. Still images required in the test section were cropped versions of images used for the video.

The full experiment consisted of six sessions of fMRI data acquisition, the learning phase constituted the third and fourth acquisition. During the experiment subjects learnt the five gestures with their associated sounds, they were tested on each association twelve times during the process of learning.

Learning was behaviourally measured using an analogue scale which was operated by the right hand, i.e., the same hand used for gesturing. An impression of the forty one point rating scale is provided by figure 1, (top right). No numbers were shown on the screen just an increasing or decreasing number of coloured blocks. The scale could run from left-to-right or right-to-left depending on whether the correct answer was a ‘match’ between sound and gesture or not. Subjects were trained in the use of this scale prior to data acquisition using task irrelevant judgements on statements accompanied with appropriate images, such as “the president thinks he has won”, “the monkey is excited”. At the onset of the rating session the cursor origin was always centrally placed. Subjects were explicitly asked to give accurate rather than rushed responses. For each test trial, the “sound only” was played followed by a jittered interval (3.5 s+/−1.5 s) where only a fixation cross was presented. Then the visual analogue scale was presented. After completion of operating the scale a jittered inter stimulus interval (3.5 s+/−1.5 s) followed prior to beginning either the next test trial or next learning block. The action components were thus temporally separated from the event of interest, i.e., “sound only”, to remove confounds of the action.

Upon hearing the tone, a preparatory motor response for using the scale could vary in accord with learning, thus confounding the analysis. Scale usage was therefore designed so that a single button press was required to start the scale, the response cursor then moved along the scale automatically step by step. A second button press stopped it. This broke the control of the scale into distinct movements. The subject did not prepare the amount of time they had to hold down the button, but only which of two buttons they had to press to start moving along the scale in the direction of their desired response. This should therefore not have had a parametric impact upon the ‘sound only’ event which stimulated it.

**Figure 2:**
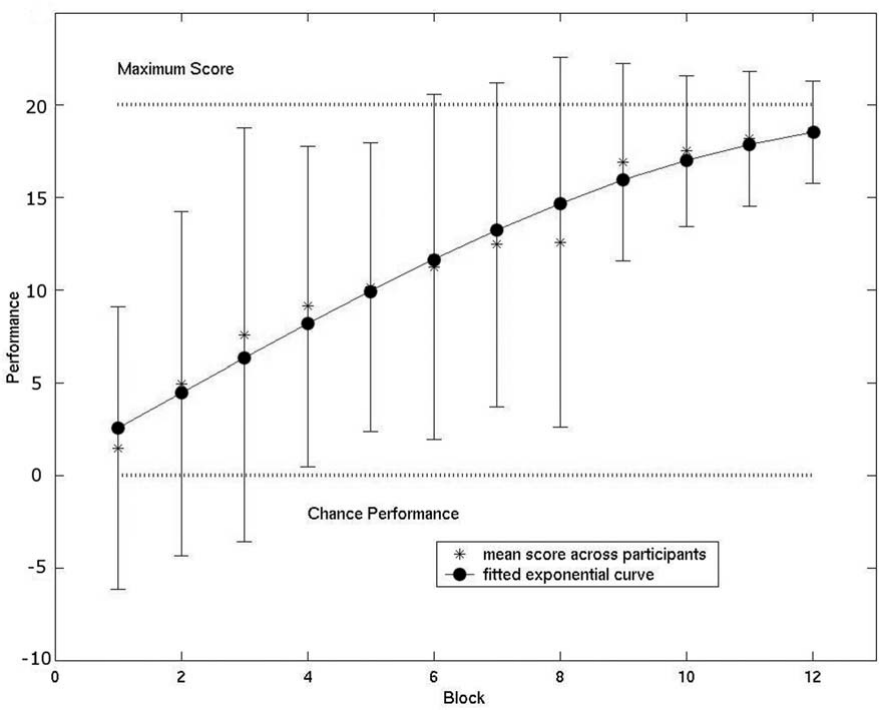
Behavioural data shows increase in learning from chance to maximum. Error bars equal one standard deviation from mean, a fitted exponential curve to the behavioural data was used to identify brain regions correlated to learning. The fitted curve was used as the input to identify changes in BOLD signal to sound only events correlated with learning.

During testing blocks, subject's responses as to whether sound and gesture match were collected on the forty one point visual analogue scale, (−20∶20). Scores of 1∶20 represented correct responses with increasing confidence. A score of 0 indicated *‘no idea’* what was the correct response, whilst a score of 20 indicated a correct response with full confidence. Scores −20∶−1 represented false alarms with decreasing confidence of being correct, i.e. −20 indicated full confidence of being correct but actually being incorrect. Scores for each block (5 judgements) were averaged within each subject. Figure 2 shows the mean and one standard deviation from the mean of these scores across participants in blue. MATLAB 6 (The Mathworks Inc) was used to fit a curve to the behavioural data using a second degree logarithmic model (seen in red, Figure 2). This curve was used for inputting parametric values to the sound events during the experiment. The curve was re-sampled to give 60 values as opposed to the original 12 so that learning within a block was modelled as smoothly increasing during blocks rather than increasing stepwise from block to block.

The other data acquisitions were all oddball tasks in which either ‘sound only’ or ‘video only’ were presented to the participants. Each of the oddball data acquisitions took approximately 3.5 min. The prior presentation of the sounds and videos familiarized the subjects with the stimuli but did not create associations.

Scanning was conducted on a 3T system (Siemens Trio) with a gradient echo EPI T2* sensitive sequence, using a standard head coil. Contiguous gradient echo, echoplanar images in 42*3 mm slices no gap, with interleaved acquisition, TR 2450 ms, TE 20 ms, flip angle 80° were acquired. Slices covered the entire brain positioned parallel to the plane intersecting the anterior and posterior commissure. The matrix acquired was 64×64 with a FOV of 192×192 mm^2^. High-resolution (1×1×1 mm voxel size) T_1_-weighted structural MRI was acquired for each volunteer using a three dimensional FLASH sequence. Timing of stimuli and temporal logging of push button responses in relation to data acquisition were controlled from a separate PC using “Presentation” (www.neurobs.com). During the first session which consisted of 6 blocks of learning and training, the mean number of volumes acquired per subject was 319.9+/−11.0 (mean, standard error). The second half, (a further 6 blocks) was slightly shorter due to reduced response times, (mean number of volumes acquired per subject = 298.0+/−9.6). The overall duration of the associative learning data acquisitions was therefore approximately 25 minutes for each subject.

Imaging data was pre-processed and analysed using SPM2 with the data series realigned to the first volume, normalized to MNI standard space (interpolating to 3 mm cubic voxels) and smoothed using a Gaussian kernel of 9 mm full width half maximum prior to conducting event related analysis.

For parametric analysis the following four event types were entered as regressors modeled with a canonical hemodynamic response function (cHRF). (1) ‘sounds with video of gesture events’ (presented in learning phases). (2) ‘sound events presented alone’, (presented in test phases). (3) ‘copy seen gesture event’, (presented in association phases). (4) button presses (required during test phases). The cHRF of the 60 type (1) and type (2) events was modulated parametrically using the learning curve derived from behavioural data (as described above). The key contrast was the main effect of the parametric modulation of the ‘sound only’ events of the test phases. Each participant's contrast image was used in a second-level analysis (t-tests) treating participants as a random effect. As we had a strong a-priori hypothesis focused upon BA44, a mask created from the cytoarchitectonic maps of BA44 [31] was applied which included all voxels with >50% probability of being from region BA44, (maps available from www.bic.mni.mcgill.ca/cytoarchitectonics). The posterior parietal mask was based on the combined superior and inferior parietal regions as defined by the automatic anatomical labelling (aal) template [32] implemented through the WFU Pick atlas (http://www.fmri.wfubmc.edu/) software [33], [34]. We report data from these small volumes combined into a single mask and thresholded at P<0.05 FWE corrected. We also report whole brain analysis results thresholded at P<0.001 uncorrected. Two thresholds are used as the effects within the BA44 and IPL are the main focus of our hypothesis and we wish to highlight the strength of the result. Other data is presented as additional observations to allow the reader to judge for themselves our interpretation of the data.

**Figure 3:**
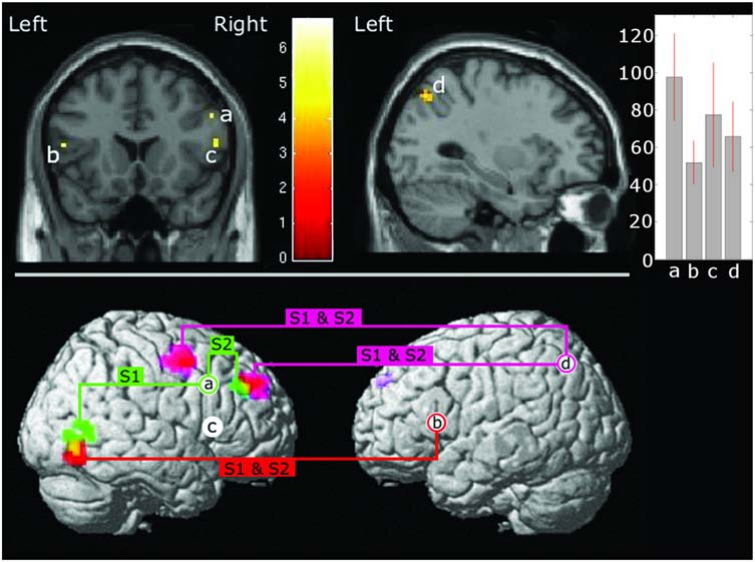
Parametric and connectivity imaging results. Top Panel: Primary Parametric Analysis: Coronal slice y = 18. Negative correlations to learning are seen in both left and right BA44, (a) xyz = 51 15 36, Z = 4.19, (b) xyz = −51 18 12, Z = 3.56, (Broca's Region) (c) xyz = 57 18 12, Z = 3.35. As well as in the inferior parietal lobule, shown on sagital slice x = −30, (d) xyz = −30 −69 48. Images generated at P<0.05 FWE and small volume corrected. Contrast of estimates & 90% confidence interval for each cluster given in bar chart on right. Lower Panel: Secondary Functional Connectivity Analysis: Four seed regions of interest identified by our initial parametric analysis (top panel) are marked as white circles. Clusters from PPI connectivity analysis derived from each seed are rendered onto the single subject MNI template brain with the colour coded key, red = left BA44 analysis; green = right BA44 analysis (dorsal); magenta = left inferior parietal lobule analysis. Yellow colouring in a cluster denotes locus of colocalization from two separate PPI analyses. “S1 & S2” indicates effects were seen across both sessions, “S1”/“S2” indicates the effect was observed in session 1or session 2 only.

**Figure 4:**
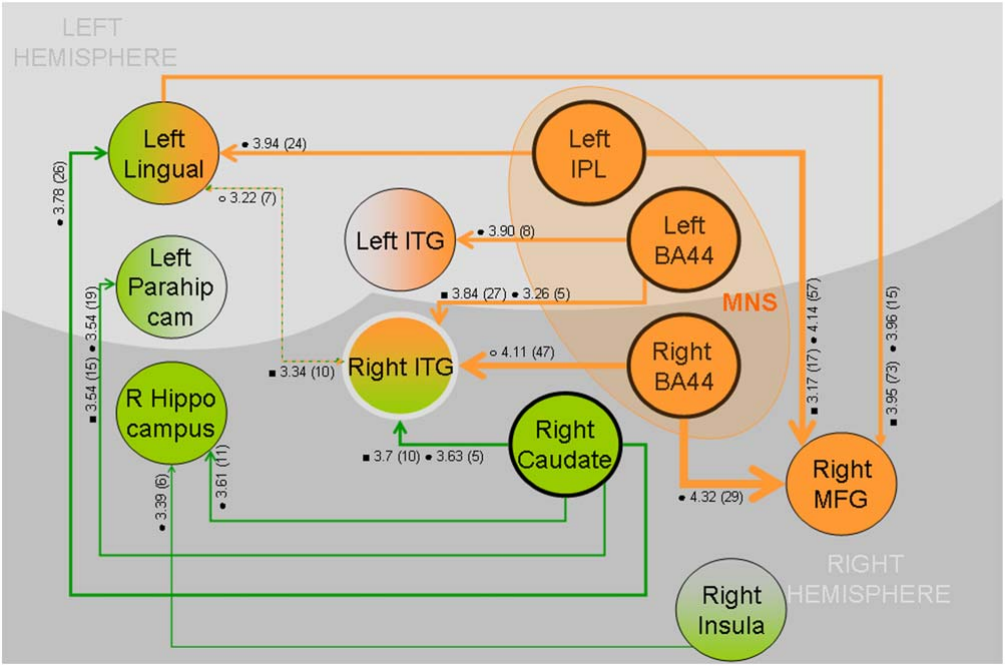
Increasing connectivity as a function of learning between regions identified by initial parametric analysis. PPI analysis (P<0.001 uncorrected, voxel size> = 5) was conducted using each coordinate in Table 1 as a seed, (highest peak within 6 mm of coordinate for individual subject's seed). Here we show results where one seed showed connectivity within 8 mm of a second seed. The direction of the arrow indicates the direction of analysis. The direction of the arrow does not indicate causality. Empty circle = effect found in session 1, filled circle = effect found in session 2, filled square = effect found across sessions. Value following symbol = Z score, value in parenthesis = cluster size. i.e., analysis of right insula as seed identified a cluster (6 voxels; Z = 3.39) showing increasing connectivity with learning proximal to the right hippocampus in second session.

Secondary functional connectivity (Psychophysiological interaction) analysis was conducted after successful (parametric) analysis of fMRI data. By extracting the time course from a seed voxel (physiological factor) and multiplying it with the learning curve (psychological factor) the interaction of the seed voxel's activity with learning was derived. This was then re-implemented as a regressor of interest into a general linear model with the two predictors of the interaction as regressors of no interest. Data was subjected to analysis and statistical parametric maps displaying regions indicative of the psychophysiological interaction produced. To test for the hypothesis of an involvement of the MNS in this task, bilateral BA44 and the single cluster from the posterior parietal region were chosen as seeds of interest for the PPI analysis (Figure 3–top panel). Nevertheless an analysis was also conducted on all other peak loci (Table 1a) derived from whole brain analysis for parametric responses to learning (see Figure 4 for these additional results). The full method [35] consisted of extracting the fMRI time series and deconvolving the seed's BOLD signal to gain an approximation of the underlying neuronal signal. Seeds were derived by finding the individual's peak point from within a 6mm radius of the second level analysis coordinate. The underlying neuronal signal was then multiplied by the learning curve to express an interaction between the two. Finally, the approximation was reconvolved with a standard hemodynamic response function to provide a regressor modelling regions that show a BOLD response demonstrating the interaction of seed region with learning. Again, results of whole brain analysis are reported at P<0.001 uncorrected cluster size> = 5, yet we point specifically to the areas in which we observe co-localizations from a multi PPI approach (Figure 3). This threshold suffices to eliminate speculation that effects observed in the primary parametric analysis are an artifact due to non-specific reductions in BOLD signal.

**Table 1:** 1a Negative Correlations between BOLD response to Sound Only Events and Learning.

Anatomical Location	Left Hemisphere					Right Hemisphere				
			mni coordinates					mni coordinates		
	BA	Z Score	x	y	z	BA	Z Score	x	y	z
Orbital Frontal Gyri	-	-	-	-	-	BA47	4.63	36	39	−12
Insula	-	-	-	-	-	BA47	4.39	27	21	−12
**Frontal Operculum (Broca's)**	**BA44**	**4.32**	**−48**	**18**	**15**	**BA44**	**4.19**	**51**	**15**	**36**
**Inf Temp Gyrus (Area V5)**	BA37	4.22	−45	−54	−9	**BA37**	**4.28**	**57**	**−48**	**−12**
Head of Caudate	-	-	-	-	-	-	4.27	15	15	12
Hippocampus	-	-	-	-	-	-	4.05	27	−24	−21
Para Hippocampus Gyrus	BA36	3.8	−18	−39	−12	-	-	-	-	-
Lingual Gyrus	BA37	4.16	−18	−81	3	-	-	-	-	-
**Caudal Intraparietal Sulcus**	**BA7**	**3.83**	**−30**	**−69**	**48**	-	-	-	-	-

Primary Parametric Analysis: Tabled regions showing negative correlation of BOLD response to sound events as a function of learning. Whole brain analysis, threshold p<0.001 uncorrected, cluster size>10. Those regions in bold type also showed increasing functional connectivity as a function of learning in the secondary functional connectivity analysis.

Contrasts between responses to sound and video pre- and post-learning were also created. The pre-processed imaging data of pre-learning sound sessions and post-learning sound sessions were analysed using a general linear model. Regressors were implemented separately for sound events and oddball events for each session after a high pass filter of 127.5 Hz was applied to remove low frequency artefacts in the data. In order to identify differences in BOLD signal between the ‘sound only’ events in both sessions, a t-test was conducted. Subsequently, t values were transformed into Z scores. The same analysis was applied to the video events of the pre- and post-learning sessions.

## Results

The analysis of behavioural data demonstrated a clear improvement in performance from chance and high error rates to almost perfect performance and low error rates (Figure 2). In the primary parametric analysis, the fitted logarithmic curve displayed in Figure 2 was used to model the expected learning related changes to the BOLD response occurring as a result of ‘sound only’ events. The top panel of figure 3 shows highly significant, negative correlations between the BOLD response and the behavioural data in our regions of interest (ROI), i.e., BA44 bilaterally and l-IPL, in the second level analysis. The interpretation of this result is that the BOLD signal elicited by sound only stimuli reduces in power as behavioural performance improves. It should be noted that this is not a negative BOLD response, but a parametric decrease in activity of a positive BOLD response. Note, that the bilateral STG regions (auditory cortex) remained comparably activated to sound stimuli throughout the learning phase, indicating that the effects in BA44 are not due to simple suppression or lower arousal. Moreover it can be ruled out that a BOLD decrease simply occurs as an effect of time [36]. Right BA44 territory stretches considerably more dorsally than left BA44 territory. This is particularly evident when viewing the right BA44 clusters ‘a’ and ‘c’.

Besides the analysis of ROI, we also conducted full brain analysis which revealed additional negative correlations, bilaterally in the inferior temporal gyrus, and right-hippocampus as well as in other regions (see Table 1a for full results). These regions therefore appeared to mirror the reduction in activity as a function of increasing performance. There were no significant findings at the chosen threshold (P<0.001, uncorrected) for positive correlations with performance.

Videos and sounds were presented independently in sessions both before and after the learning sessions. We did not anticipate considerable effects in direct contrasts between these sessions as each sound-action had only been practiced/viewed on 12 occasions. In previous studies which conduct such direct contrasts, hours of practice are typically used [28], [37]. The main effect of ‘video stimuli post-learning’ however did reveal additional clusters of activation not seen pre-learning. This included Broca's region (l-BA44) and the posterior parietal regions as expected (see Supplementary Material ‘Results S1’). It is not contradictory that activity reduces during the learning task yet shows increased activity when comparing post to pre learning sessions. In the pre-learning session, subjects are unaware of the upcoming task and have no reason to process the gesture as something to imitate or to have linkage to sound. In the post-learning session they now understand the linkage, have practiced the gesture and process accordingly thus with increased activation in BA44. Statistical comparison between sessions yielded limited areas where significant differences could be identified (see Supplementary Material ‘Results S1’).

As a secondary step, we employed a psychophysiological interaction (PPI) analysis to identify brain regions with BOLD signals that show an interaction between the BOLD signal from a seed i.e., l-BA44 (the physiological factor) and performance (the psychological factor). Positive results identify regions with increasing functional connectivity with the seed as a function of performance. Analysis was first conducted using each of the three clusters identified within BA44 and the l-IPL as seeds in accord with our hypothesis. Each seed in BA44 was analysed individually as the functional anatomy of BA44 predicts that each cluster will conduct a differing component of the task [38]. Secondly, this analysis was conducted from all other peak point loci, which showed a correlation between learning and BOLD response but were not part of our original hypothesis (given in Table 1a). Results of particular interest were regions that show this relationship to more than one seed region. Co-localizations of such types demonstrate that an actual ‘network’ has been identified rather than a group of commonly activated clusters.

The results of connectivity analyses on BA44 and l-IPL seeds are presented in the bottom panel of Figure 3, which shows regions (greater than 10 voxels) which are functionally connected to two separate seeds. Two regions fulfill this criterion of co-localization from two ROI seeds. For both sessions, increasing connectivity was displayed between both l-BA44 and r-BA44 to a cluster, which straddles the right-middle-occipital-gyrus and the right-inferior-temporal-gyrus (r-ITG). In the first session of learning this increasing connectivity predominated between right BA44 (cluster a) and an area localized at r-ITG *(x,y,z = 51, −69, −12; Z = 4.1)*. Present continuously throughout both sessions was an increased connectivity between l-BA44 (cluster b) and another area localized at r-MOG *(x,y,z = 52, −74, 3; Z = 3.8)*, slightly more ventral than the r-ITG region. As can be seen in the lower panel of Figure 3, these connected areas co-localize around an area *(x,y,z = 53, −70, −6)* which lies ventral to and possibly overlapping with visual area V5 as described previously [39].

Connectivity with the right-hemisphere BA44 (cluster a) in the second session was towards the right-middle-frontal-gyrus *(x,y,z = 39, 33, 33; Z = 3.8)*. This region co-localized with a region from the PPI analysis of the l-IPL seed. This parietal seed showed increasing connectivity to both the middle frontal gyrus region *(x,y,z = 30, 39, 33; Z = 4.1)* and the dorsal premotor cortex *(x,y,z = 51, 0, 51; Z = 4.14).* Co-localization centre of the two middle frontal gyrus clusters was at *(x,y,z = 32, 39, 32)*. The other seed region in the right BA44 (cluster c) did not reveal connectivity with other regions in the second session and at the given thresholds. In Figure 4, we show a cartoon summary of all the connectivity analyses conducted (P<0.001 uncorrected, cluster size> = 5). This shows two distinct ‘ends’ to the network, one encapsulated by the concept described as the MNS (orange), the other by regions typical of learning and memory (head of caudate and hippocampus, green). The head of caudate, l-BA44, r-BA44, r-ITG, l-IPL all showed considerable connectivity to within 8 mm of other seed regions. Notably, neither the right-caudate, albeit with numerous other connections, nor the right-hippocampus showed any connectivity to the MNS seed regions and vice versa. However, MNS seeds and hippocampus/caudate seed shared connectivity to the visual perception areas of r-ITG and lingual gyrus.

Correlation analysis between time courses of BA44 area and l-IPL (within MNS) were compared to correlations between MNS and ‘other’ regions using appropriate t-tests of Fisher-transformed-Pearson's R values [40]. This tested the hypothesis that the absence of increasing connectivity within the MNS components was due to existing high levels of connectivity making a significant difference due to learning difficult to observe. It also allowed us to rule out that lack of observed connectivity between regions such as the hippocampus and MNS regions may also be due to this same ceiling effect. Correlation of time course was greater between MNS regions than between MNS components and hippocampus (P<0.001) but not between MNS regions and caudate (P = 0.12), (See Supplementary Materials ‘Results S1’).

## Discussion

The aim of the present fMRI study was to investigate whether BA44 is involved in the formation of links between sounds and gestures. We addressed this issue by two approaches. First, we evaluated the correlation of learning gesture-sound associations with the BOLD signal. Second, we applied a functional connectivity analysis in order to a) remove the possibility of the results in the parametric analysis being a confound of strategy or non-specific effects of reduction in BOLD signal, b) to identify further brain areas, which are connected to BA44 and are incorporated in the dynamic process of learning sound-gesture associations.

The first analysis showed BOLD signal decreases as a function of learning of sound-gesture associations bilaterally in BA44. The results illustrate very clearly the process of repetition suppression [41], [42] occurring within a learning paradigm, highlighting the involvement of BA44 for this task. Multiple other regions showed similar behaviour during this analysis however, leading to a question of whether effects were causal or merely downstream effects due to an auditory-visual association occurring elsewhere, i.e., the hippocampus. Indeed, the largest effect in this first analysis was observed in the right orbital frontal gyrus, also a site taking in processed information from across multimodal streams. Given the results of the first analysis we can only conclude that our regions of interest are involved in gesture-sound association learning but not that they orchestrate it.

The second analysis displayed that l-BA44, r-BA44, r-ITG, l-IPL, left-hippocampus, right-head-of-caudate, r-MFG and premotor cortex combine to form a network which increases its connectivity as a function of learning a sound-gesture association. Clearly, regions that show increasing connectivity as a function of learning in tandem with decreases in BOLD amplitude also correlated to learning are intimately involved in the learning process. Regions such as the orbital frontal gyrus did not show this kind of dual effect, allowing us to focus more accurately upon key regions. Yet the caudate and hippocampus did. We are unable to distinguish causal effects given the current paradigm. However, if our results were downstream effects of learning then we would anticipate connectivity between regions such as the hippocampus/caudate with the MNS. On the contrary however, there is no significant connectivity with these regions, moreover the effects seen in relation to the MNS are considerably higher than those seen emanating from analysis of other regions (Figure 4).

The correlation analysis between the performance data of learning of sound-gesture associations and the BOLD signal revealed a learning related decrease of activity (not deactivation) of left and right BA44 and the l-IPL. These regions comprise the so called “mirror neuron system” (MNS). A well documented system for matching action perception with action execution, as well as action recognition [9], [10], [43]. In several studies it has been shown that decreasing BOLD activation occurs with learning [44], [45]. This also applied for the classical speech regions [46], where activation negatively correlated with success in phonetic learning. The authors suggest that this is due to more efficient processing. Indeed, such results illustrate a process akin to repetition suppression [41], [42], which indicates that a neural network required to encode and process the stimuli becomes sparser, but more efficient by elimination of redundant activity. Accordingly, a reduction of BOLD signal is an expected outcome of learning [47]. The negative correlation between learning and BOLD signal as identified in the present study can therefore give additional evidence for the presence of actual learning.

It has to be mentioned that we anticipated greater activation in the MNS due to sound post-learning compared to pre-learning. Increased activation was observed between sessions but did not pass threshold. However, compared to learning novel, more finely tuned movements such as playing guitar chords [28], or piano sequences [37] our task requires minimal effort and is only practised briefly in the scanner. Possibly the lack of statistically significant increased activation post-learning is a consequence of not having engaged the system with a sufficiently demanding motor-learning component of the task as well as having only 12 practice events between sessions for each gesture. Our experiment is designed to identify dynamic effects of learning, not upon identifying the ‘pre versus post’ consequences of learning. The power of the experiment lies within the limited exposure to stimuli before pairing and capturing the ‘process of acquisition’, not ‘consolidation’ of learning. The results of our study only carry an implication for the ‘acquisition stages’ of learning the association; not mediation of the learned activity itself.

Interestingly, the identified learning related decrease of activity in the left and right BA44 and the l-IPL was not related to an increase or decrease in connectivity between these areas. However, significant increased connectivity was identified with other brain regions, such as r-ITG, r-MFG, and right-premotor-cortex, all of them connected to at least two MNS areas. An even larger number of areas displayed increased connectivity with one MNS region, these included the left ITG and the lingual gyrus, therefore comprising areas of perception and working memory, suggesting a mediating role for the MNS regions. Both caudate and hippocampus show involvement in learning identical in nature to that of the MNS yet do not appear to be linked to the MNS. Perhaps these regions serve highly generalized components of associative learning whilst the MNS is more strongly related to those with a strong motor component.

As the key event, the ‘sound only’ condition was analysed. It was presented to the subject prior to the test question, “Does the sound match the gesture in the picture?”. The subject's task at this time point may be split into two components: First, recognising the sound together with its action association, and second, holding the sound-gesture information in memory until the test picture is presented. Indeed, both of these task contents are reflected in the connected areas.

Let us first focus upon the action-sound recognition component. For action-sound recognition we would expect to find regions linking together typically involved in the recognition of learnt actions and sounds. These should include the MNS based gesture recognition, as well as sound and vision recognition. In the present study those are represented by the areas of bilateral BA44 and bilateral ITG. These areas show increased connectivity in either both sessions or in the first one, reflecting the temporal process of encoding and recognition. Left BA44 (Broca's area) is best known for both covert and overt speech production [38] but also for the production of complex hand movements [2], [48]. Parts of l-BA44 do not only comprise oro-facial representations but also representations for finger and hand movements as it displays activation in either task (for a recent meta-analysis of functions of Broca's region see the work of Lindenberg [49]). Right BA44 is a good candidate for undertaking the association between hand movements and tones as it is known to be commonly activated when studying the tonal aspects of tonal languages [50], [51]. Activations in the r-ITG are often generated by observing body parts or point light biological motion [52], [53]. Nevertheless, the peak voxel of the cluster in this study lay more ventro-caudal as compared to previous studies, who reported the maxima roughly dorsal to area V5. This, as well as the lack of activation in the multi-sensory regions of the superior temporal sulcus sensitive to human movement [54]–[56] might be due to the stimuli not having common features across modalities [57]. However, V5 activations were also reported in the study by Puce [56] during which subjects observed eye and mouth movements. Moreover, the elegant ‘imitation’ study of Makuuchi did focus on accurately imitating hand gestures instead of the common posture imitations. Strong activations of the whole r-ITG area including and surrounding V5 were observed [58]. In fact, even when symbolic cues with a short delay before performance were used to elicit the gesture, this area was still activated.

Taking together the functions of l-BA44, r-BA44 and the r-ITG, we therefore propose that these regions constitute a true network encoding the multi-sensory stimulus–a true network insofar as the increased connectivity between them indicates their increased binding together to become the neural signature for the combined stimulus. The parallel processing of the hippocampus is not discounted, however the connectivity profiles on the whole suggest that this is a separate process most likely sub-serving an audio-visual associative learning component of the task.

Secondly let us focus on the working memory component expected to be elicited during the key event. For the memory component of the task, one would expect to find motor working memory areas and indeed, the connectivity analysis displayed a strong learning-related network between r-MFG, l-IPL, and r-BA44. The MFG is consistently activated during working memory tasks [59] and plays a primary role in memory storage [60]. The detected increased functional connectivity of the MFG with the region being involved in tonal processing (r-BA44) as well as with the region constituting the MNS parietal component (IPL) supports the concept of MFG's role in motor working memory processes. The finding that the connectivity is especially pronounced in the second session emphasizes it even further.

Why, however, does the parietal component of the MNS show this relationship rather than the ventral pre-motor component of the MNS? It has been suggested that two parallel dorsal visual streams may exist: a dorso-dorsal stream and a ventro-dorsal stream, which pass through the superior and inferior parietal lobules, respectively [61], [62]. The dorso-dorsal stream supposedly mediates immediate, online actions feeding into the dorsal pre-motor cortex. The ventro-dorsal stream, which mediates more complex visuospatial information and has higher working memory capacity, delivers information into the vPMC. As sound and gesture become associated, the interaction between memory storage in the MFG, holding the ‘sound’, is increased with the IPL motor working memory regions of the motor system holding the ‘gesture’. MNS theory posits that the MNS recalls the gesture associated to a sound by resonating with the movement by means of implicit internal simulation. Indeed, it was not uncommon for the subjects to have the urge to make a slight left hand movement during the presentation of sounds. The right hand used for imitating was occupied with the button box at this point. This hand movement is what may be expected in light of work showing sympathetic muscle activity. This kind of muscle activity has been reported for several stimulus and muscle groups, e.g., in viewing actions or hearing action words [63]. As hearing speech elicits muscle activity in the tongue muscles [17]. Similarly, words associated with actions specific to particular body parts such as ‘kick’ and ‘tackle’ or ‘thump’ and ‘grab’ elicit muscle activity within the appropriate limb [20]. This phenomenon of sympathetic muscle activity can therefore supplement the explanation of increasing connectivity found between l-IPL and the right-hemisphere pre-motor system.

More precisely, we suggest that the IPL component of the MNS is involved in gesture recall and replay by accessing the stored sounds from the MFG, which receives processed sound input from r-BA44.

There are caveats of MNS as an emerging network involved in gesture-sound associations. Taking together the ties of the previously described action-sound recognition network and the memory network, the MNS appears to be involved in mediating these associations in humans. A secondary system involving the caudate and hippocampus exists yet they seem less strongly involved and although they interact with the same visual regions do not appear connected to the MNS components themselves. Connectivity data is reported uncorrected for multiple comparisons. It is used primarily to counter claims that the strong effect of reducing BOLD activity with learning identified in the parametric analysis is an artefact. It should also be noted that we use the acronym MNS with some reservation. These regions are known to house mirror neurons yet they share many other types of neurons and we cannot categorically assign effects in these areas directly to such neurons. Also, we note a complete lack of significant connectivity between regions supposedly part of a ‘system’. One would assume that components of a system should become more functionally connected during learning. This is a question of great interest and so far we can only suggest that connectivity is consistently high between these regions and that achieving a significant change in that level of connectivity is difficult to image. Correlations in time courses were significantly greater between l-IPL and BA44 than those between BA44 and the hippocampus, suggesting this may be the case. However, these results are not substantial enough to be complete. Finally, we acknowledge that this experiment does not show new stimuli being integrated into a form of communication, only that new stimuli can be linked to the motor system in a way that *could* allow them to be communicated.

In summary we show that left and right Brodmann Area 44 and left-intra-parietal-lobule are part of an emerging network during the learning of novel sound-gesture associations. The data suggests these regions reduce their BOLD activity as learning progresses yet increase their connectivity to visual processing and working memory regions. This data demonstrates that the brain regions thought to comprise the Mirror Neuron System in the human are indeed involved in the linking of novel sounds and gestures. These brain regions appear to work in parallel with other memory and associative-learning brain regions which also show connectivity to the same visual processing and working memory regions. This data does not demonstrate that the associative system we see is used for communication, only that it is there, available for use.

## Supporting Information

Results S1 Document showing addition data.
(0.55 MB DOC)

Video S1 Stimuli used for experiment.
(21.49 MB AVI)
