# Supplementary material for: Neural Dynamics of Learning Sound—Action Associations
Source: PLoS One. 2008 Dec 3;3(12):e3845. doi: 10.1371/journal.pone.0003845 (PMC2585816; doi:10.1371/journal.pone.0003845)
Supplement: Results S1 — Document showing addition data. (0.55 MB DOC) [file pone.0003845.s001.doc]

**Supplementary Results:**


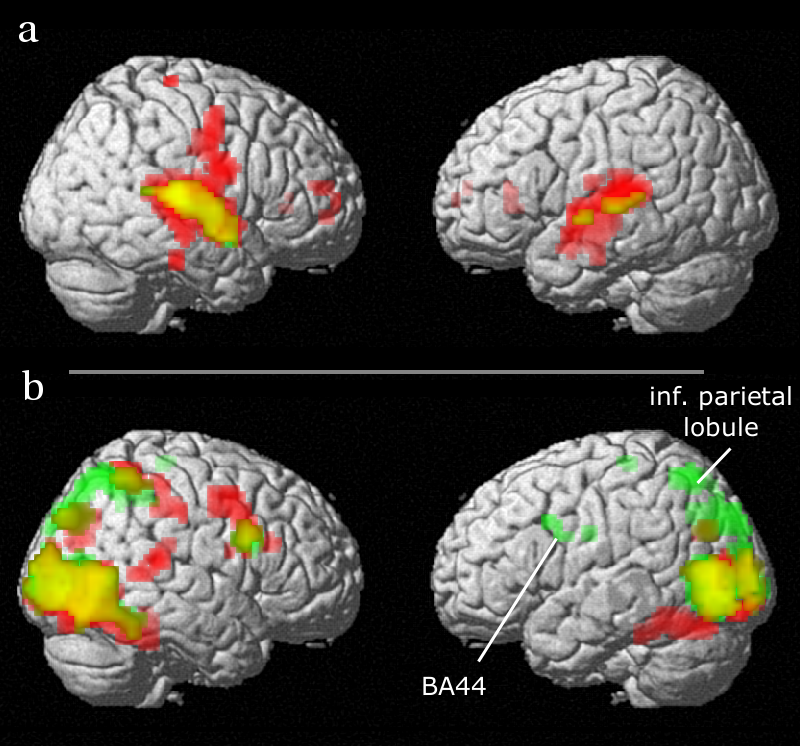


**Figure 1**. a) Main effect of sounds rendered upon canonical MNI single subject brain. b) Main effects of video rendered upon same brain. Red = pre-learning; Green = post-learning; Yellow = Activation during both pre and post learning. All four SPM’s calculated at P < 0.001 uncorrected, cluster size > 10. Note green clusters in Broca’s area (left BA44) and left inferior parietal lobule when viewing videos.

Supplementary Table

| **Table 1b Effects of direct contrasts of stimuli before (s1) and after (s2) learning.** | | | | | | |  |
| --- | --- | --- | --- | --- | --- | --- | --- |
|  |  |  |  |  |  |  |  |
| **Contrast** | **Anatomical Location** | **BA** | **# vox** | ***Z* score** | ***x*** | ***y*** | ***z*** |
| Sound s1 > Sound s2 | Left Fusiform | 48 | 13 | 3.96 | -33 | -18 | -24 |
| Sound s1 > Sound s2 | Left Insula | 48 | 14 | 3.94 | -39 | -9 | 0 |
| Sound s1 > Sound s2 | Cerebelum 6L | - | 10 | 3.93 | -24 | -42 | -33 |
| Sound s1 > Sound s2 | Left Paracentral Lobule | 4 | 10 | 3.55 | -8 | -33 | 51 |
| Sound s2 > Sound s1 | No Significant Clusters |  |  |  |  |  |  |
| Video s1 > Video s2 | Right Insula | 6 | 13 | 3.76 | 48 | -3 | 36 |
| Video s2 > Video s1 | Left Paracentral Lobule | 4 | 13 | 4.08 | -3 | -30 | 66 |

Results from contrasting elicited by sound only or video only stimuli before learning (s1) or after learning (s2). Whole brain analysis, threshold p < 0.001 uncorrected, cluster size > 10. MNI = Montreal Neurological Institute, BA = Brodmann Area, #vox = cluster size.


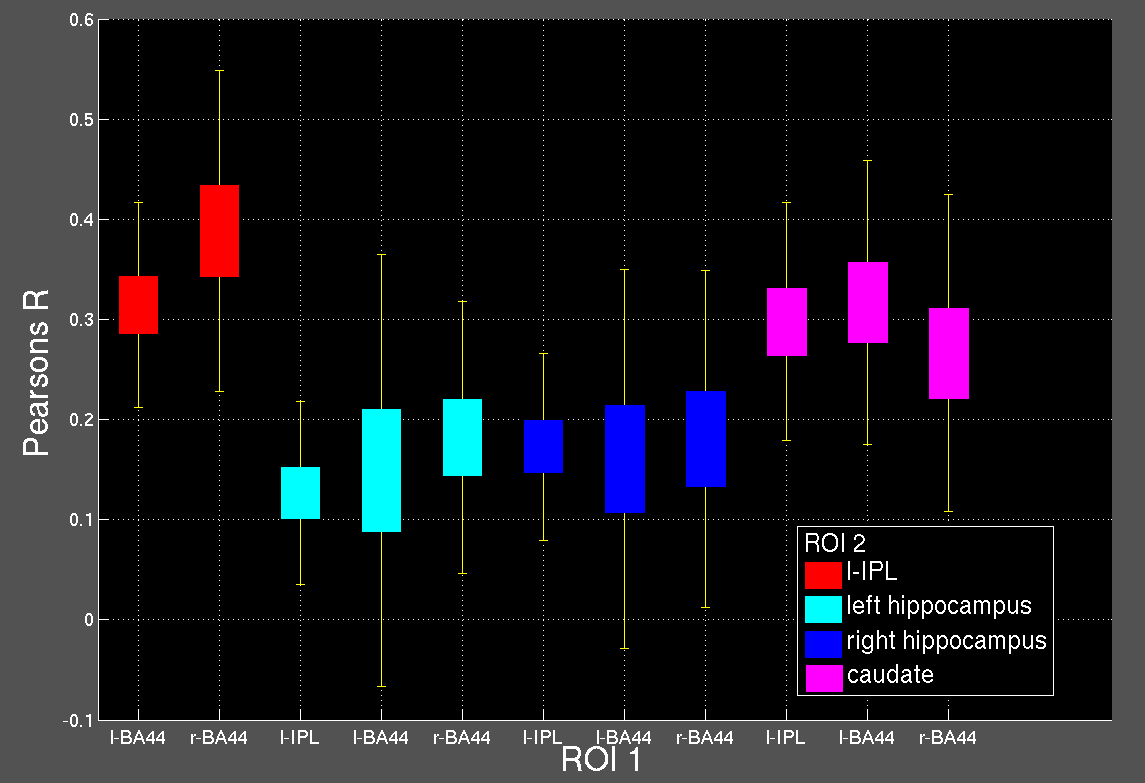


**Figure2:**

Analysis was conducted similarly to that described previously (McNamara et al., 2007). Time courses from ROI’s were extracted for each subject and the Pearson’s Correlation coefficient calculated between pairs. The graph shows standard error of mean (coloured patch) and standard deviation (error bars) of correlations between ROI 1 (x axis) and ROI 2 (see legend) during Session 1 of the experiment. There were no significant differences between sessions so session one is used to illustrate. A random sample of 200 time courses taken from grey matter over 40mm apart yielded a baseline level correlation of R=0.167, (+/- 0.03). Red patches indicate within MNS regions and show high levels of correlation throughout the experiment. These high levels of connectivity may mask learning related activity between regions of the MNS in the experiment. Cyan and blue patches show significantly lower (see below) correlations between hippocampal ROIs and MNS ROIs. These values are very similar to baseline levels of correlation. Therefore it is unlikely that effects of connectivity due to learning are masked. Caudate however showed similar levels of correlation of time course with MNS as seen between regions of the MNS. Therefore we cannot exclude the possibility that effects of learning on connectivity of the caudate with the MNS are masked.
